# Effects of perceived spiritual management, work engagement, and organizational commitment on job satisfaction among clinical nurses: the mediating role of perceived spiritual management

**DOI:** 10.1186/s12912-023-01625-x

**Published:** 2023-12-06

**Authors:** Eun-Hye Lee, Hea-Jin Yu

**Affiliations:** College of Nursing, Sahmyook University, 815, Hwarang-ro, Nowon-gu, Seoul, 01795 Republic of Korea

**Keywords:** Job satisfaction, Spirituality, Management, Hospital, Nurses

## Abstract

**Background:**

Spirituality in the workplace has a positive impact on organizations. It strengthens employees’ well-being and their quality of life. It also gives work a sense of purpose and meaning and creates a sense of interconnectedness.

**Methods:**

This study is a descriptive cross-sectional survey which intends to determine how job satisfaction is impacted by spiritual management, work engagement, and organizational commitment from the perspective of clinical nurses. Responses to self-administered questionnaires were collected from 230 hospital nurses in South Korea from July to August, 2022.

**Results:**

Job satisfaction was positively correlated with perceived spiritual management (r = .662), job commitment (r = .514), and organizational commitment (r = .587). Perceived spiritual management had the highest correlation with job satisfaction, followed by organizational commitment and job commitment. To determine the effect of these factors on clinical nurses’ job satisfaction, a hierarchical multiple regression analysis-in other words, a method that controls the entry order of a series of independent variables-was conducted. Model 4 ultimately explained 58.4% of job satisfaction (R^2^ = 0.584; F = 44.563; *p* < .001), with an additional 2.5 explained variance. Among the control variables in Model 4, only marital status (β = 0.173) was positively significant; perceived spiritual management (β = 0.388), work engagement (β = 0.208) and organizational commitment (β = 0.225) were all found to have significant positive effects on job satisfaction. The mediation analysis showed that perceived spiritual management had a partial mediating effect on the relationship between work engagement, organizational commitment, and job satisfaction.

**Conclusion:**

The results of this study confirm that job satisfaction for nurses requires not only individual predispositions, but also active changes in management strategies, such as spirituality management at the hospital’s organizational level.

## Introduction

Among the many essential elements within hospital organizations, the nursing position plays a central role, since it generally involves spending a great deal of time with patients and acting as one of the key contributors toward patients’ healthcare. Thus, effective management of nurses is of the utmost importance in improving the quality of nursing services [1, 2]. Nurses’ job satisfaction is highly linked to nurse turnover rates, which can negatively impact the quality of nursing services if there is a labor shortage [2]. The nurse turnover rate in hospitals is notably high in Korea, averaging 13.9%, significantly surpassing the overall industry turnover rate of 4.8% during the same period. Particularly noteworthy is the turnover rate among newly recruited nurses, which stands at 29%, indicating a significantly elevated proportion [3]. Within the highlights of job satisfaction that is known to work as an index of nurse retention, some of the well-known factors that are linked to job satisfaction. Studies show that job engagement [4], organizational commitment [5], and spiritual management [6] are the crucial factors that impact job satisfaction in the workplace.

First of all, job engagement is one of the most important factors that is related to job satisfaction. The exact opposite of “job burnout,” job engagement, refers to the degree of emotional commitment, enthusiasm, and involvement, that employees have toward their work and their organization [7]. Engaged employees are fully ambitious and passionate about their duties, feel a strong connection to their organization’s goals and values, and are willing to go the extra mile to contribute to the potential success of their workplace [8]. Job engagement is also, the essence of the relationship between an organization and an individual, where a highly dedicated employee desires to exist as a member of a specific organization, competes with great effort to achieve the organization’s benefits, and embraces the goals and values pursued by the organization, holding a clear conviction about them, which leads to job satisfaction [9].

Along with job engagement, organizational commitment is highly linked to job satisfaction. Organizational commitment is lexically defined as a strong emotional attachment, dedication, and a willingness to remain with an organization [10]. In essence, it refers to the attitude of members within an organization who embrace its goals and values, placing their efforts toward the organization’s growth and success. Organizational commitment also becomes dependent upon external conditions and internal motivation [11]. The results of the study conducted by Hedayat et al. (2018) indicate a strong and meaningful connection between organizational commitment and job satisfaction. The regression coefficient reveals that organizational commitment accounts for 42.2% of the variations in job satisfaction [12].

Moreover, perceived spiritual management is another extremely important factor related to job satisfaction. It is defined as a management style that goes beyond merely pursuing the interests of a single organization and considers the well-being of our society as a whole [4]. Perceived spiritual management emphasizes the existing business values of cost reduction, productivity improvement, efficiency enhancement, and customer satisfaction while fostering community communication, cooperation, and coexistence. Job engagement and organizational commitment are very closely related to spiritual management which is connected to job satisfaction [13, 14]. In this regard, perceived spiritual management can be viewed as the mediator that may explain how job engagement and organizational commitment influence of job satisfaction [15].

Along with spiritual management, job satisfaction is essential for every individual and every organization, particularly nurses, as it is a determinant index of the retention of nurses in clinical settings [16]. Moreover, beyond the evident significance of job satisfaction, research has shown that contented employees enjoy improved health and extended lifespans. Additionally, job satisfaction tends to positively influence an employee’s life beyond their professional sphere [12].

Many investigators have conducted research studies regarding job satisfaction and the factors related to it. Nevertheless, most of these studies have focused on non-medical personnel, such as human resource employees [17], bank employees [18], and employees in educational sectors [6].

Furthermore, summarizing the relevant literature reveals that job engagement has an impact on spiritual management [13] and job satisfaction [8]. Spiritual management has been shown to have a positive impact on job satisfaction [4]. Additionally, organizational commitment influences spiritual management [19] and job satisfaction [10]. However, existing studies have primarily focused on the correlations among these variables, and there is limited research on the specific pathways through which spiritual management affects the relationship between job engagement/organizational commitment and job satisfaction, particularly in the context of clinical nursing. Therefore, this study aimed to focus on the job satisfaction of clinical nurses and investigate the mediating effect of perceived spiritual management in the relationship between their job engagement, and the mediating effect of perceived spiritual management between organizational commitment and job satisfaction. By doing so, it sought to provide foundational data for the development of a program that promotes increased job satisfaction among clinical nurses through the enhancement of spiritual management.

### Purpose

The purpose of this study was to investigate the impact of job satisfaction on nurses in clinical settings. The specific aims were: (1) to assess the level of perceived spiritual management, job engagement, organizational commitment, and job satisfaction; (2) to explore the correlations between perceived spiritual management, job engagement and organizational commitment among the participants; (3) to assess direct effects of perceived spiritual management, work engagement, and organizational commitment on job satisfaction; (4) to assess the mediating effect of perceived spiritual management in the relationship between job engagement and job satisfaction; and (5) to assess the mediating effect of perceived spiritual management in the relationship between organizational commitment and job satisfaction.

### Research hypotheses

#### Hypothesis 1

The level of job engagement is positively associated with job satisfaction.

#### Hypothesis 2

The level of job engagement is positively associated with spiritual management.

#### Hypothesis 3

The level of spiritual management is positively associated with job satisfaction.

#### Hypothesis 4

The level of organizational commitment is positively associated with job satisfaction.

#### Hypothesis 5

The level of job engagement is positively associated with spiritual management.

## Methods

### Study design

This study is a descriptive cross-sectional survey to determine the impact of spiritual management, job engagement, and organizational commitment on job satisfaction as perceived by current clinical nurses in Korea.

### Research subjects

The participants in this study were clinical nurses working in more than 10 general hospitals in Seoul and Gyeonggi provinces, both of which have a large number of general hospitals in Korea. The number of subjects was calculated using the G*Power 3.1.9 program for regression analysis with a significance level of ⍺ of 0.05, a power of 95%, an effect size of 0.15, and 14 independent variables (4 independent variables and 10 characteristics of the subjects), resulting in a minimum sample size of 194. Based on the dropout rate of previous studies related to job satisfaction of clinical nurses [20–22], the data collection was conducted considering an incomplete response rate of 20%. 240 copies were distributed, and a total of 230 copies were returned for final analysis. Therefore, the sample size used in this study can be considered adequate for statistical power.

### Research instruments

#### Perceived spiritual management

Spiritual management is defined as a management that leads to the engagement and enthusiasm of employees by respecting the capabilities and participation of individuals, and furthermore, by respecting the life satisfaction and happiness of individuals, while thinking about society as a whole and not just pursuing the interests of a single company. Spiritual management was measured using an index developed by Lee Jung-ah and Seo Yong-won [23] that can diagnose the level of spiritual management practice in an organization. The Spiritual Management Index consists of 40 items in seven sub-factors: social responsibility (5 items), spiritual leadership (7 items), decentralized organization (3 items), fair personnel system (9 items), work-life balance (8 items), member growth (4 items), and transparent organizational culture (4 items). The scale is based on a 5-point Likert scale ranging from ‘strongly disagree’ (1) to ‘strongly agree’ (5), with higher scores indicating higher spiritual management practices. At the time of tool development, the internal reliability was Cronbach’s ⍺ value of 0.88 [23], and in this study, it was 0.97.

#### Job engagement

The Utercht Job engagement Scale (UWES) instrument developed by Schaufeli and Bakker [24] and translated by Shin et al. [25] was used with permission. The UWES is a 17-item 5-point Likert scale with three subscales: vitality (6 items), motivation (6 items), and commitment (5 items), ranging from 1 “strongly disagree” to 5 “strongly agree,“ with higher scores indicating higher job engagement. At the time of development, the overall Cronbach’s α of the instrument was 0.93, and in Kim’s study, the Cronbach’s α was 0.92 [26], and 0.93 in this study.

#### Organizational commitment

Organizational Commitment Organizational commitment was measured using the Organizational Commitment Questionnaire (OCQ) developed by Mowday et al. [27] translated into Korean by Park [28] and validated for reliability and validity by Joo et al. [29] for nurses. The instrument has a total of 15 items and is composed of three sub-factors: 5 items of identification, 5 items of organizational attachment, and 5 items of work motivation. Each item is answered on a 5-point Likert scale ranging from 1 (strongly disagree) to 5 (strongly agree), and the measured score ranges from 15 to 75, with higher scores indicating higher organizational commitment. In Mowday et al’s [27] study, the reliability, Cronbach’s ⍺ value, was 0.89, in Joo’s study [29], Cronbach’s ⍺ value was 0.86, and in this study, it was 0.94.

#### Job satisfaction

An instrument developed by Slavitt et al. [30] and translated by Park, Sung-Ae and Yoon, Soon-Nyeong [31] was modified and supplemented by Lee, Ho-Kyung [32] to measure nurses’ job satisfaction. The instrument consists of 40 items and includes autonomy, relationships, compensation, work demands, professional pride, and organizational needs. Each item was measured on a 5-point rating scale, with 5 ‘being always true’, 4 ‘being often true’, 3 ‘being only true’, 2 ‘being rarely true’, and 1 ‘being never true’, and negative items were reverse scored. Higher scores indicate higher job satisfaction. The reliability of Cronbach’s ⍺ in Slavitt et al.‘s [30] study was 0.91, and Cronbach’s ⍺ in this study was 0.93.

### Data collection and ethical considerations

This study was approved by the Institutional Review Board (IRB no. SYU 2022-02-014) of the researcher’s institution, S University, to ensure the ethical aspects of the study, and data collection was conducted in July and August 2022. Before the survey, the questionnaire was revised and supplemented by consulting with both professors of nursing management and field experts on the appropriateness of the survey questions and research subjects. To determine the appropriateness of the questions and the ease of understanding, three clinical nurses were asked to provide their opinions on the questionnaire. For data collection, the researcher visited the nursing department of the target medical center to explain the purpose and method of the study and to obtain their approval and cooperation for data collection. Nurses who agreed to participate in the study were provided with an informed consent form stating that the data would not be used for any purpose other than the research, that they could cancel their participation at any time during the study, and that all data would be anonymized and kept confidential.

### Data analysis

The data of this study were analyzed using SPSS/WIN 25.0 program. The general characteristics of the study subjects and hospital job-related characteristics, perceived spiritual management, job engagement, organizational commitment, and job satisfaction were analyzed using descriptive statistics. Cronbach’s α value was used for the reliability of the measurement tools.

Differences in perceived spiritual management, job engagement, organizational commitment, and job satisfaction according to the general characteristics of the subjects were analyzed by independent t-test, ANOVA, and post hoc analysis by Scheffe test. The correlation between perceived spiritual management, job engagement, organizational commitment, and job satisfaction was analyzed by Pearson’s correlation coefficient.

Hierarchical Multiple Regression was used to analyze the effects on job satisfaction of the subjects. For the analysis, the variables of gender, marital status, religion, and work experience, which had a statistically significant difference in job satisfaction, were analyzed as dummy variables based on less than two years. In addition, to check the mediating effect of the remaining research variables on the relationship between the variables with the greatest explanatory power on job satisfaction in the multiple regression analysis, a mediation effect analysis was conducted by applying Model 4 of SPSS PROCESS Macro proposed by Hayes [33]. To determine the statistical significance of the indirect effects, 10,000 bias-adjusted boot strapping with 95% confidence interval was performed.

## Results

### Differences in perceived spiritual management, job engagement, organizational commitment, and job satisfaction according to the general characteristics of the study subjects

The results of the analysis of the differences in perceived spiritual management, job engagement, organizational commitment, and job satisfaction according to the general characteristics and characteristics of the research subjects are shown in Table 1. A total of 230 subjects participated in the study, with 91.9% being female, 67.8% being single, and 71.7% being four-year graduates. In addition, 55.2% were between the ages of 20 and 29, and 77.8% had more than three years of clinical experience.

**Table 1:** Difference in Spiritual Management, Job engagement, Organizational Commitment and Job Satisfaction according to General Characteristics of the Participants (N = 230)

Characteristics	Categories	n(%)	Perceived Spiritual Management		Job engagement		Organizational Commitment		Job Satisfaction	
			M ± SD	t/F *(p*)	M ± SD	t/F *(p*)	M ± SD	t/F *(p*)	M ± SD	t/F *(p*)
Gender	Male	21(9.1)	3.38 ± 0.75	1.50(0.222)	3.29 ± 0.77	5.79(0.017)	3.92 ± 1.35	1.89(0.171)	3.22 ± 0.43	4.66(0.032)
	Female	209(90.9)	3.21 ± 0.56		2.94 ± 0.63		3.58 ± 1.03		3.00 ± 0.44	
Age(Year)	20∼29^a^	127(55.2)	3.34 ± 0.57	6.26(0.002) a > b, b < c	2.87 ± 0.61	7.84(0.001) a,b < c	3.56 ± 1.06	5.08(0.007) a,b < c	3.09 ± 0.43	3.49(0.032)
	30∼39^b^	74(32.2)	3.05 ± 0.58		2.98 ± 0.67		3.49 ± 0.98		2.92 ± 0.46	
	≥over 40^c^	29(12.6)	3.19 ± 0.52		3.39 ± 0.61		4.18 ± 1.12		3.01 ± 0.41	
Marital status	Married	74(32.2)	3.09 ± 0.62	6.41(0.012)	3.13 ± 0.70	6.88(0.009)	3.76 ± 1.07	2.03(0.156)	2.90 ± 0.43	8.32(0.004)
	Singe	156(67.8)	3.29 ± 0.55		2.90 ± 0.60		3.54 ± 1.05		3.08 ± 0.43	
Education	Associate degree^a^	53(23.0)	3.22 ± 0.61	0.22(0.800)	2.81 ± 0.65	7.45(0.001) a,b < c	3.47 ± 1.09	1.64(0.196)	3.03 ± 0.46	0.85(0.428)
	Bachelor’s degree^b^	165(71.7)	3.22 ± 0.56		2.98 ± 0.61		3.63 ± 1.02		3.01 ± 0.43	
	≥Master degree^c^	12(5.3)	3.34 ± 0.80		3.58(0.83)		4.07 ± 1.41		3.18 ± 0.47	
Religion	Religious	81(35.2)	3.30 ± 0.57	2.18(0.142)	3.14 ± 0.61	8.53(0.004)	3.89 ± 1.09	8.63(0.004)	3.16 ± 0.44	12.10(0.001)
	None	149(64.8)	3.19 ± 0.58		2.88 ± 0.66		3.46 ± 1.02		2.95 ± 0.42	
Working Department	Medical word^a^	67(29.2	3.14 ± 0.61	1.49(0.227)	2.90 ± 0.68	3.58(0.029) a,b < c	3.57 ± 1.12	0.16(0.856)	3.01 ± 0.50	0.11(0.893)
	Surgical word^b^	102(44.3)	3.30 ± 0.53		2.91 ± 0.61		3.60 ± 0.95		3.04 ± 0.42	
	Special word^c^*	61(26.5)	3.21 ± 0.63		3.16 ± 0.65		3.67 ± 1.19		3.02 ± 0.41	
Working type	Shift	185(80.4)	3.27 ± 0.58	4.76(0.030)	2.89 ± 0.64	15.43(< 0.001)	3.54 ± 1.03	4.35(0.038)	3.04 ± 0.45	1.27(0.262)
	Fixed	45(19.6)	3.06 ± 0.55		3.30 ± 0.60		3.91 ± 1.13		2.96 ± 0.41	
Position	Staff nurse	187(81.3)	3.26 ± 0.59	2.40(0.123)	2.91 ± 0.62	10.70(0.001)	3.51 ± 1.07	9.73(0.002)	3.04 ± 0.44	0.61(0.437)
	Nurse manager	43(18.7)	3.10 ± 0.51		3.26 ± 0.69		4.06 ± 0.93		2.98 ± 0.43	
Career(year)	1∼≤2	51(22.2)	3.61 ± 0.56	16.58(< 0.001) b,c < a	3.02 ± 0.71	1.25(0.288)	3.87 ± 1.02	3.06(0.049)	3.27 ± 0.44	11.19(< 0.001) b,c < a
	3∼≤5	68(29.6)	3.15 ± 0.46		2.87 ± 0.51		3.39 ± 1.01		2.99 ± 0.35	
	>5	111(48.2	3.10 ± 0.58		3.01 ± 0.69		3.63 ± 1.09		2.93 ± 0.45	
Type of hospital	≥ 500	94(40.9)	3.21 ± 0.53	0.21(0.646)	2.94 ± 0.60	0.40(0.526)	3.60 ± 0.95	0.02(0.883)	2.98 ± 0.42	1.85(0.175)
	<500	136(59.1)	3.24 ± 0.62		3.00 ± 0.68		3.62 ± 1.14		3.06 ± 0.45	

a,b,c,d Alphabet is the result of a post hoc test using Scheffé’s method

*Special word: operation room, intensive care unit, emergency room

According to the general characteristics of the subjects, awareness of spiritual management was higher among nurses aged 20–29 (3.34 ± 0.57) and 40+ (3.19 ± 0.52) than among nurses aged 30–39 (3.05 ± 0.58) (F = 6.26, *p* = .002) shift nurses (3.27 ± 0. 58) than fixed-duty nurses (3.06 ± 0.55) (t = 4.76, *p* = .030) and nurses with less than 2 years of experience (3.61 ± 0.56) perceived higher than nurses with 3–5 years of experience (3.15 ± 0.46) and more than 5 years of experience (3.10 ± 0.58) (F = 16.58, *p* < .001).

Analyzing differences in job engagement according to the general characteristics, males (3.29 ± 0.77) were higher than females (2.94 ± 0.63) t = 5.79, *p* = .017), nurses in their 40s and older (3.39 ± 0.61) were higher than nurses in their 20–29 years (2.87 ± 0.61) and 30–39 years (2.98 ± 0.67) (F = 7.84, p=. 001), married nurses (30.1 ± 0.70) were higher than unmarried nurses (2.90 ± 0.60) (t = 6.88, *p* = .009), nurses with a master’s degree or higher (3.58 ± 0.83) were higher than nurses with a diploma (2.81 ± 0.65) and bachelor’s degree (2.98 ± 0.61) (F = 7. 45, *p* = .001), nurses with religion (3.14 ± 0.61) were higher than nurses without religion (2.88 ± 0.66) (t = 8.53, *p* = .004), and nurses working in special departments (3.16 ± 0.65) were higher than nurses working in internal medicine wards (2.90 ± 0.68) and surgical wards (2. 91 ± 0.61) than those working in the medical ward (F = 3.58, *p* = .029), nurses working full-time (3.30 ± 0.60) were higher than those working shifts (2.89 ± 0.64) (t = 15.43, *p* < .001), and nurses who were junior managers or above (3.26 ± 0.69) were higher than general nurses (2.91 ± 0.62) (t = 10.70, *p* = .001).

The differences were analyzed for organizational commitment, nurses aged 40+ (4.18 ± 1.12) were higher than nurses aged 20–29 (3.56 ± 1.06) and 30–39 (3.49 ± 0.98) (F = 5.08, *p* = .007), and nurses with religion (3.89 ± 1.09) were higher than nurses without religion (3. 46 ± 1.02) than those with no religion (t = 8.63, *p* = .004), full-time nurses (3.91 ± 1.13) were higher than shift nurses (3.54 ± 1.13) (t = 4.35, *p* = .038), and junior nurses (4.06 ± 0.93) were higher than general nurses (3.51 ± 1.07) (t = 9.73, *p* = .002).

According to the general characteristics of the subjects, job satisfaction was higher among males (3.22 ± 0.43) than females (3.00 ± 0.44) (t = 4.66, *p* = .032), single (2.90 ± 0.43) than married (3.08 ± 0.43) (t = 8.32, *p* = .004), and nurses with religion (3. 16 ± 0.44) were higher than nurses with no religion (2.95 ± 0.42) (t = 12.10, *p* = .001), and nurses with less than 2 years of experience (3.27 ± 0.44) were higher than nurses with 3–5 years of experience (2.99 ± 0.35) and more than 5 years of experience (2.93 ± 0.45).

### Correlations between subjects’ perceived spiritual management, job engagement, organizational commitment, and job satisfaction and variables

The perceived spiritual management, job engagement, organizational commitment, and job satisfaction of the study subjects are shown in Table 2. The mean of perceived spirituality was 3.23 ± 0.58 on a 5-point scale, job engagement was 2.97 ± 0.65, organizational commitment was 3.61 ± 1.06, and job satisfaction was 3.02 ± 0.44.

**Table 2:** Descriptive statistics and Correlations of Spiritual management, job engagement, organizational commitment, Job satisfaction (n = 230)

Variables	a	b	c	d	Min	Max	M ± SD	Skewness	Kurtosis	AVE	CR
	r(*p*)	r(*p*)	r(*p*)	r(*p*)							
Spiritual Management(a)	1				1.55	4.90	3.23 ± 0.58	0.13	0.21	0.966	0.995
Job engagement(b)	0.403(< 0.001)	1			1.24	5.00	2.97 ± 0.65	0.02	0.12	0.967	0.988
Organizational Commitment (c)	0.536(< 0.001)	0.640(< 0.001)	1		1.17	6.44	3.61 ± 1.06	0.12	-0.21	0.950	0.982
Job Satisfaction(d)	0.662(< 0.001)	0.514(< 0.001)	0.587(< 0.001)	1	1.70	4.48	3.02 ± 0.44	0.30	0.83	0.956	0.992

*AVE = average variance extracted; CR = composite reliability*

The correlations between the research variables are shown in Table 2. Job satisfaction, the dependent variable of this study, was correlated with all variables. Job satisfaction was positively correlated with perceived spiritual management (r = .662), job engagement (r = .514), and organizational commitment (r = .587). Among them, perceived spiritual management had the highest correlation with job satisfaction, followed by organizational commitment and job engagement.

Additionally, in order to test convergent validity, the average variance extracted (AVE), and composite reliability (CR) were used. In our study, all criteria (AVE: >0.5, CR: >0.7) were met (Table 2). In addition, all item loadings were statistically significant and exceeded a value of 0.5. Regarding discriminant validity, all of the square roots of AVE for each construct were greater than its highest correlation with any other construct.

### Effects of perceived spiritual management, Job Engagement, and organizational commitment on job satisfaction

To determine the effects of perceived spiritual management, job engagement, and organizational commitment on job satisfaction among clinical nurses, a hierarchical multiple regression analysis was conducted, an analysis method that controls the order of entry of a series of separate independent variables. The results of the hierarchical regression of the outcome variable, of job satisfaction, on the general characteristics of gender, marital status, religiosity, experience, perceived spiritual management, job engagement, and organizational commitment are shown in Table 3. The Durbin-Watson statistic for the regression analysis was 2.133, which assumes the independence of the error terms. Regarding multicollinearity, the multicollinearity indexes among the independent variables are 0.854 for perceived spiritual management, 0.904 for job engagement, and 0.926 for organizational commitment, which is less than 1.0, and the Variance Inflation Factor (VIF) is 1.170 for perceived spiritual management, 1.106 for job engagement, and 1.080 for organizational commitment, which shows that there is no problem of multicollinearity.

**Table 3:** Hierarchical multiple regression for job satisfaction

Variables	Model 1				Model 2				Model 3				Model 4			
	B	SE	β	t(p)	B	SE	β	t(p)	B	SE	β	t(p)	B	SE	β	t(p)
constant	3.484	0.229		15.227(0.000)	1.949	0.223		8.730(0.000)	1.333	0.230		5.781(0.000)	1.364	0.225		6.073(0.000)
Sex	-0.199	0.093	-0.131	-2.142(0.033)	-0.148	0.074	-0.097	-2.010(0.046)	-0.075	0.069	-0.049	-1.084(0.280)	-0.077	0.068	-0.050	-1.137(0.257)
Marital Status	0.138	0.059	0.147	2.349(0.020)	0.084	0.047	0.089	1.785(0.076)	0.149	0.045	0.159	3.335(0.001)	0.163	0.044	0.173	3.723(0.000)
Religion	-0.223	0.056	-0.243	-3.973(0.000)	-0.166	0.045	-0.180	-3.712(0.000)	-0.125	0.042	-0.137	-2.990(0.003)	-0.113	0.041	-0.123	-2.759(0.006)
Career(year)	0.256	0.066	0.242	3.871(0.000)	0.048	0.055	0.046	0.873(0.384)	0.074	0.051	0.070	1.447(0.149)	0.074	0.050	0.070	1.477(0.141)
Spiritual Management					0.458	0.039	0.606	11.692(0.000)	0.353	0.040	0.466	8.761(0.000)	0.294	0.042	0.388	6.920(0.000)
Job engagement									0.214	0.035	0.316	6.104(0.000)	0.141	0.040	0.208	3.566(0.000)
Organizational commitment													0.093	0.026	0.225	3.640(0.000)
F(p)	11.681(< 0.001)				42.324(< 0.001)				47.190(< 0.001)				44.563(< 0.001)			
${R}^{2}/ \text{a}\text{d}\text{j}. {R}^{2}$	0.172(0.157)				0.486(0.474)				0.559(0.548)				0.584(0.571)			
${R}^{2}$change	0.172				0.314				0.074				0.025			

The hierarchical regression analysis regressed the four general control variables of gender, marital status, religious affiliation, and work experience on job satisfaction in the first step (Model 1), perceived spiritual management in the second step (Model 2), job engagement in the third step (Model 3), and organizational commitment in the fourth step (Model 4). Model 1, which included only four control variables, was statistically significant and explained 15.7% (R2 = 0.157, F = 11.681, *p* < .001) of job satisfaction, with all four control variables having a significant impact, but being married (β = 0.147) and having two or fewer years of experience (β = 0.242) had a positive impact. In Model 2, which controls for general characteristics and includes perceived spiritual management, job satisfaction was explained by 47.4% (R2 = 0.474, F = 42.324, *p* < .001), and perceived spiritual management explained an additional 31.4% of job satisfaction. Model 3, in which job engagement was added to the control variables and perceived spiritual management, explained a total of 55.9% (R2 = 0.559, F = 47.190, *p* < .001) of job satisfaction, an additional 7.4 explanatory power. Model 4, which included organizational commitment, ultimately explained 58.4% of job satisfaction (R2 = 0.584, F = 44.563, *p* < .001), an additional 2.5 explanatory power. Among the control variables in Model 4, only marital status (β = 0.173) was positively significant, and perceived spiritual management (β = 0.388), job engagement (β = 0.208), and organizational commitment (β = 0.225) were all found to have a significant positive effect on job satisfaction.

### The mediating effect of perceived spiritual management on the relationship between job commitment and job satisfaction of organizational commitment

To test the mediating effect of perceived spiritual management, which has the greatest explanatory power among the variables explaining job satisfaction in the relationship between clinical nurses’ job commitment and organizational commitment in the hierarchical regression, the results of the analysis using model 4 of PROCESS macro proposed by Hayes [33] are shown in Table 4.

**Table 4:** Significance of the mediation effect analysis of Spiritual management in the relationship between job satisfaction job engagement, organizational commitment

Direct effect	β	SE	t	p	95% CI	
					LLCI	ULCI
JE→SM	0.374	0.052	7.117	< 0.001	0.2704	0.4774
	R = .551 R^2^ = 0.303 F = 19.493 *p* < .001					
JE →JS	0.214	0.035	6.104	< 0.001	0.1449	0.2831
SM→JS	0.353	0.040	8.761	< 0.001	0.2735	0.4322
	R = .748 R^2^ = 0.559 F = 47.190 *p* < .001					
OC→SM	0.282	0.030	9.522	< 0.001	0.2238	0.3406
	R = .626 R^2^ = 0.392 F = 28.861 *p* < .001					
OC→JS	0.139	0.023	6.153	< 0.001	0.0947	0.1839
SM→JS	0.316	0.043	7.342	< 0.001	0.2313	0.4010
	R = .749 R^2^ = 0.560 F = 47.382 *p* < .001					
**Indirect effect**	**Effect**		**Boost SE**		**95% CI**	
					**LLCI**	**ULCI**
JE →SM→JS	0.195		0.029		0.0803	0.1943
Total Effect	0.346		0.067		0.2738	0.4181
OC→SM→JS	0.089		0.018		0.0567	0.1284
Total Effect	0.229		0.021		0.1867	0.2703

*Note: Control variables were sex, marital status, religion and career*

*JE = Job engagement; OC = organizational commitment; SM = spiritual management; JS = Job satisfaction*

*CI = confidence interval; LLCI = lower limit confidence interval; SE = Standard error; ULCI = Upper limit confidence interval*

Before testing the mediating effect of perceived spiritual management, we checked the assumptions of the regression analysis and found that the intercept of the skewness was not greater than 3, and the size of the kurtosis was not greater than 10 (Table 2), so all variables met the normal distribution. Through regression analysis, the linearity of the model, normality of errors, and homoscedasticity were confirmed, and there was no multicollinearity problem due to the Durbin-Watson index value and the marginal value of the tolerance. To verify the significance of the mediating effect of perceived spiritual management on the effects of job commitment and job engagement on job satisfaction of clinical nurses, this study conducted a bootstrap with 10,000 repeated sampling. In addition, gender, marital status, religious affiliation, and work experience, which showed significant differences in job satisfaction variables among the general characteristics of the study subjects, were corrected as control factors.

**Fig. 1:**
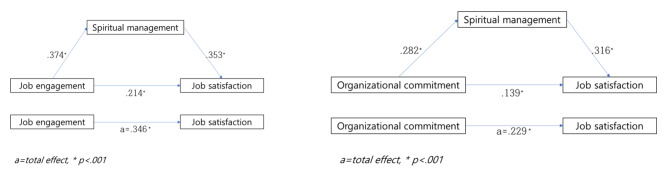
Causal relationship between variables by the PROCESS Macro

When analyzing the direct effects of job commitment and perceived spiritual management on the relationship between clinical nurses’ job commitment and job satisfaction, clinical nurses’ job commitment had a positive effect on the mediator, perceived spiritual management (β = 0.374, *p* < .001), and the explanatory power of the model was 30.3%. Clinical nurses’ job commitment (β = 0.214, *p* < .001) and perceived spiritual management (β = 0.353, *p* < .001) had a direct effect on clinical nurses’ job satisfaction, with an explanatory power of 55.9%. When the significance of the mediating effect of perceived spiritual management on the effect of clinical nurses’ job commitment on job satisfaction was tested, the indirect effect was 0.195 with a confidence interval of 95% (0.0803–0.1943), and the bootstrap upper and lower values did not include zero, confirming the mediating effect of perceived spiritual management (Fig. 1).

When analyzing the direct effects of job commitment and perceived spiritual management on the relationship between clinical nurses’ organizational commitment and job satisfaction, clinical nurses’ job commitment had a positive effect on the mediator, perceived spiritual management (β = 0.282, *p* < .001), and the explanatory power of the model was 39.2%. Clinical nurses’ organizational commitment (β = 0.139, *p* < .001) and perceived spiritual management (β = 0.316, *p* < .001) had a direct effect on clinical nurses’ job satisfaction, with an explanatory power of 56.0%. When the significance of the mediating effect of perceived spiritual management on the effect of clinical nurses’ job enthusiasm on job satisfaction was tested, the indirect effect was 0.089 with a confidence interval of 95% (0.0567–0.1284), and the bootstrap upper and lower values did not include zero, confirming the mediating effect of perceived spiritual management (Fig. 1).

## Discussion

This study was a descriptive, correlational research design on 230 clinical nurses with more than one year of experience to examine the mediating effect of perceived spiritual management on the relationship between job engagement and job satisfaction, and on organizational commitment and job satisfaction. As numerous pieces of literature emphasize, hospitals must bear in mind that the effective management of nurses plays a crucial role in enhancing the quality of nursing services. Job satisfaction among nurses is closely linked to nurse turnover rates, which can have adverse effects on nursing services in the presence of labor shortages [2]. Increased job satisfaction can lead to higher nurse retention rates [1, 2]. When experienced nurses remain in their positions, it fosters improved continuity of patient care, as these nurses have a greater familiarity with patient needs and hospital procedures [3]. Additionally, contented nurses are more likely to engage in efficient communication with both patients and colleagues. Effective communication constitutes a fundamental aspect of delivering high-quality patient care, ensuring the accurate sharing and understanding of information within the healthcare team [34]. To the best of our knowledge, this is the first research effort on job satisfaction among clinical nurses, and perceived spiritual management being a mediator. The discussion based on the main results is as follows:

First of all, in the first step (model 1) of hierarchical regression analysis, four control variables (gender, marital status, religion, and year of career) had a regression effect on job satisfaction among clinical nurses. This is consistent with previous studies [35, 36] that reported significant differences in job satisfaction levels based on common characteristics. Therefore, it is likely that nurses who are male, single, have religion, and have had fewer years in their career will tend to have higher job satisfaction than those who are not. Especially regarding career years as a clinical nurse, the average score for job satisfaction in our study was 3.02 out of 5.00, and more than half of the participants had more than five years of nursing experience. This finding is similar to previous studies that, Park [37] conducted and stated that 254 head nurses from six hospitals with over 200 beds had a mean job satisfaction score of 2.73 out of 5.00. Also, Seo [38], conducted research in two medical institutions under the same foundation, with data from 348 nurses, most of them being supervisory nurses and the mean was found to be 2.93. It could be explained that the long period of repetitive nature of emotional labor lowers job satisfaction and increases turnover intentions, eventually leading to actual turnover. As a result, these negative effects not only impact the nurses themselves, but also have various consequences at the organizational level. It is required that diverse regular education needs to be developed in hospitals for nurses in different situations and stages of their careers.

Second, perceived spiritual management was found to be the strongest determinant of job satisfaction in clinical nurses in this study. The result is similar to prior research studies that showed factors that influence job satisfaction. In the study of Cho, & Ha [39], they confirmed that perceived spiritual management was one of the strongest factors that positively influenced job satisfaction in individuals working for community enterprises. Chen & Huang [40] stated that perceived spiritual management is a key element that determines one’s job satisfaction in diverse workplaces. In our study, perceived spiritual management has significantly increased its explanatory power significantly to 31.4%, making it a crucial variable for job satisfaction. When considering only the common control variables, the explanatory power for job satisfaction was 17.2%. However, with the inclusion of additional variables, the explanatory power increased significantly to 58.4%. This suggests that concerning nurses’ job satisfaction, factors related to the hospital’s management approach and the experiences within the workplace are more crucial than individual characteristics. Therefore, it is important that organizations develop appropriate interventions for nurses regarding enhancing positive sides of spiritual management, such as building work-life-balance and fair personal systems.

Third, perceived spiritual management acts as a mediator between job engagement and job satisfaction, and between organizational commitment and job satisfaction. The results showed that job engagement has a significant positive direct effect on perceived spiritual management, and the model accounted for 30.3% of the variance. Also, both job engagement and perceived spiritual management had direct and positive effects on job satisfaction among clinical nurses, and the explanatory power increased to 55.9% of the variance in job satisfaction.

Similarly, the results revealed that job engagement had a significant and positive direct effect on perceived spiritual management, for which the model accounted for 39.2% of the variance. Both organizational commitment and perceived spiritual management had direct and positive effects on clinical nurses’ job satisfaction; the model was 56.0% of the variance. The findings are similar to those in previous literature, which states that perceived spiritual management plays a vital role in job engagement, organizational commitment, and job satisfaction at a workplace [19, 40–42]. Consequently, evidence appears to indicate that perceived spiritual management is an extremely crucial factor that significantly boosts nurses’ job satisfaction. It suggests that as personal spirituality is underpinned, spiritual management at the institutional level (hospital) is deemed necessary.

Our study has several limitations that need to be highlighted. First, this study is limited in that it only focuses on a few general hospitals and quite a small number of participants in South Korea, making it challenging to generalize the research findings to all nursing units in healthcare institutions, when multisite approaches are needed. Additionally, the cross-sectional design of this study could limit the causal relationship between the variables. Therefore, the recommendation is to conduct large-scale, longitudinal studies that improve the limitations.

### Significance and application of Research

The academic and practical significance of this research can be summarized as follows:

#### Academic significance

This research carries academic significance as it validates the interrelationships among influential factors affecting nurses’ job satisfaction, building on prior research. It further investigates the mediating effects of spiritual management. The study’s results provide explanations and inferences regarding the concepts forming the pathways, both directly and indirectly, using the effects of these factors. Moreover, the academic importance of this research is evident in its departure from prior studies that attempted to explain spiritual management with a limited set of factors. Instead, this study examines the broader conceptual framework of the relationships and influencing factors of nurses’ job satisfaction. This expanded perspective contributes to a deeper understanding of the intricate dynamics involved in nurses’ job satisfaction, making a valuable scholarly contribution to the field.

#### Practical significance

Through this study, we were able to identify factors influencing nurses’ job satisfaction, and the variables examined in this research are mostly factors that can be intervened upon in a clinical setting with nurses. In practical terms, we believe that hospital administrators can use these results to activate spiritual management, thereby contributing to enhancing job satisfaction and the productivity of nursing tasks.

Hospitals should adopt a holistic approach, especially in the context of spiritual management (social responsibility, spiritual leadership, decentralized organization, fair personnel system, work-life balance, member growth, and transparent organizational culture), which considers both the physical and spiritual well-being of their patients and staff members [23]. Hospital leaders should promote and embody the organization’s values, fostering an environment where spirituality is acknowledged and integrated into decision-making. Also, hospital should continuously gather feedback from patients and staff regarding their spiritual experiences and use this information to improve spiritual care services [23, 39, 43]. By incorporating these strategies, hospitals can create a more holistic and compassionate healthcare environment that addresses the spiritual needs of both patients and healthcare professionals.

## Conclusions

In conclusion, these findings indicate that perceived spiritual management is the strongest factor impacting job satisfaction among clinical nurses, and plays a significant mediating role in the relationship between job engagement and job satisfaction, as well as between organizational commitment and job satisfaction among clinical nurses. Improving job engagement, and organizational commitment and fostering perceived spiritual management may lead to increased job satisfaction in this context, thereby enhancing patient care quality. On top of that, nurses’ job satisfaction is influenced more by hospital management practices and workplace experiences rather than individual characteristics. Lastly, job satisfaction serves as one explanatory variable for nurse retention; therefore, further research utilizing other dependent variables is necessary in the future.

## Data Availability

The dataset supporting the conclusions is available from the corresponding. author on reasonable request.
